# Short-chain fatty acids (SCFAs) mediated gut breast crosstalk in breast cancer: integrative network analysis of molecular signaling, immune infiltration and prognostic biomarkers

**DOI:** 10.3389/fimmu.2026.1969874

**Published:** 2026-10-28

**Authors:** Tariq Aziz, Layla A. Alahmari, Nawal H. Siddig, Mai M. Almsaud, Fahad Al-Asmari, Huda A. Alqahtani, Rasha Alonaizan, Yolande Sandrine Mengue Ngadena

**Affiliations:** 1 Molecular Microbiology and Infectious Diseases Research Unit, University of Tabuk, Tabuk, Saudi Arabia; 2 Department of Community Health, College of Applied Medical Sciences, Northern Border University, Arar, Saudi Arabia; 3 Department of Mathematical Sciences, College of Science, Princess Nourah bint Abdulrahman University, Riyadh, Saudi Arabia; 4 Department of Biology, College of Science, Imam Mohammad Ibn Saud Islamic University (IMSIU), Riyadh, Saudi Arabia; 5 Department of Food and Nutrition Sciences, College of Agricultural and Food Sciences, King Faisal University, Al-Ahsa, Saudi Arabia; 6 Department of Zoology, College of Science, King Saud University, Riyadh, Saudi Arabia; 7 Faculty of Science, King Saud University, Riyadh, Saudi Arabia; 8 Department of Animal Biology and Physiology, Laboratory of Animal Physiology, Faculty of Science, University of Yaoundé, Yaoundé, Cameroon; 9 Neurosciences axis, Laboratory of Development and Maldevelopment, Department of Psychology, Faculty of Arts, Letters, and Social Science, University of Yaoundé 1, Yaoundé, Cameroon

**Keywords:** breast cancer, gut-breast axis, immune regulation, microbial metabolites, SCFAs, stat3, therapeutic targets, tumor microenvironment

## Abstract

**Introduction:**

Breast cancer is a heterogeneous malignancy in which metabolic dysregulation, immune remodelling, and complex host microbiota interactions contribute to disease progression and therapeutic response. Emerging evidence suggests that gut microbiota-derived short-chain fatty acids (SCFAs), including acetate, propionate, and butyrate, may act as important molecular mediators linking the gut microbiome to systemic immune and tumor-associated signalling. However, the molecular networks through which SCFA-mediated signalling may influence breast cancer progression and the tumor immune microenvironment remains poorly characterized. This study aimed to investigate the potential regulatory mechanisms underlying the gut-breast axis by integrating SCFA host interaction networks in breast cancer transcriptomic, prognostic, immune infiltration, and drug gene interaction analyses.

**Methods:**

The SCFA-host interactome was constructed from the combination of HMDB and PubChem data in a protein-protein interaction network. The identification of hub genes was conducted through topological analysis, followed by validation using TCGA-BRCA RNA-seq data, Kaplan-Meier Survival Analysis, TIMER2.0 immune analysis, and DGIdb repurposing analysis.

**Results:**

The findings indicated that the genes STAT3, HIF1A, and IL6 served as key nodes connecting SCFA signalling and cancer formation. Selected SCFAs, such as propionate, have direct experimental evidence of involvement in the modulation of the JAK2/STAT3 pathway in breast cancer models, while butyrate has been associated with several breast cancer-related signalling and epigenetic mechanisms. Associations were most strongly identified in acetate which was mainly related to metabolic and immune regulatory pathways. This computational analysis did not give a quantitative ranking of the strength of interaction between SCFA and hub genes. In exploratory survival analysis without multiple-testing correction, increased STAT3 and HIF1A expression was associated with unfavourable relapse-free survival. SCFA-associated immune targets showed positive correlations with CD8+ T-cell infiltration.

**Discussion:**

This study identifies the SCFA-host interactome as an essential regulatory network in breast cancer. Uncovering metabolic and signalling chokepoints such as STAT3 leads to the discovery of new biomarkers for prognosis and drug targets. This highlights the possibility of exploiting the gut-breast axis to increase immune cell penetration.

## 1 Introduction

Breast cancer is the most commonly diagnosed cancer among women worldwide and remains a major cause of cancer-related mortality. In 2024, approximately 2.4 million women were diagnosed with breast cancer globally, with an estimated 694,000 deaths, highlighting the continuing need to better understand the molecular and systemic factors that influence disease progression and treatment response (1, 2). Although advances in molecular classification and targeted therapies have improved clinical outcomes, breast cancer remains biologically heterogeneous, and interactions between tumor cells, the immune microenvironment, metabolism, and host physiology can substantially influence the disease behavior (3). Recent research has increasingly recognized the gut microbiota as an important regulator of systemic metabolism and immunity, suggesting that microbiota-derived metabolites may contribute to cancer development and progression beyond the gastrointestinal tract (4–7).

Among these microbial metabolites, SCFAs, particularly acetate, propionate, and butyrate, have attracted considerable attention because of their ability to regulate epithelial barrier function, cellular metabolism, inflammatory responses, and systemic immunity through mechanisms involving G-protein-coupled receptors and histone deacetylase activity. Growing evidence also indicates that the gut microbiome and its metabolites may influence breast cancer biology through metabolic and immune pathways, supporting the emerging concept of the gut-breast axis (8, 9). Nevertheless, the underlying mechanisms governing the role of SCFAs in breast cancer development and management have not been comprehensively defined. Understanding these relationships would be critical to harnessing the microbiome for diagnostic and therapeutic repurposing applications (10, 11).

Accumulating evidence suggests that SCFAs act as the key messengers for the gut-breast axis, with systemic consequences arising from two principal pathways: the inhibition of HDACs and the activation of GPCRs, particularly FFAR2 and FFAR3 (12). Although the anti-proliferative role of butyrate in the development of multiple cancers via epigenetic modification leading to apoptosis has been extensively studied, the contributions of acetate and propionate to metabolic regulation and the recruitment of immune cells have gained increased attention (13). The ability of these metabolites to influence the Tumor microenvironment (TME) by modulating immune cell phenotypes for anti-tumorigenic responses has been well-established (14). In spite of the acknowledged significance of the gut-breast axis, there are still many areas that require further research, especially in terms of the systemic target network of SCFAs in breast cancer (14). To date, the vast majority of published works in this field have considered only specific pathways or molecules; thus, the overall picture of the interaction network between SCFAs and host proteins in clinical settings remains incompletely characterized. Moreover, there is an urgent need for comprehensive studies aimed at linking microbial factors to patient-level omics data, particularly their role in the maintenance of mutational stability, immune infiltrate, and survival rates (14).

The current study is relevant from both clinical and scientific perspectives since it establishes a link between microbial metabolism and precision oncology using an omics approach. In particular, the identification of the precise molecular biomarkers at which the microbiota-driven signals converge with the development of breast cancer (e.g., *STAT3* and *HIF1A*) represents a departure from correlation analysis towards a mechanistic understanding, which opens up exciting possibilities for designing targeted treatments. This study fills the gap between microbial metabolism and clinical oncology by developing a practical integrative computational framework that reveals molecular biomarkers at which gut-derived signaling interacts with breast cancer development. Despite increasing interest in microbiota-cancer interactions, the molecular networks connecting SCFA-associated host signaling with breast cancer progression, prognosis, and immune cell infiltration remains incompletely characterized. In particular, the key signaling nodes that connect microbial metabolites with oncogenic pathways and the breast cancer immune microenvironment require further investigation. Therefore, this study aimed to elucidate the regulatory networks underlying the gut-breast axis by integrating SCFA host interaction networks with breast cancer transcriptomic, prognostic, immune infiltration, and drug gene interaction analyses.

## 2 Materials and methods

### 2.1 Identification of SCFA–host target interactions

Host proteins for the major SCFAs (acetate, propionate, butyrate) were downloaded from HMDB v5.0 (https://www.hmdb.ca) and gutMGene v2.0 (http://bio-computing.hrbmu.edu.cn/gutmgene) and PubChem (https://pubchem.ncbi.nlm.nih.gov) accessed on 5^th^ April, 2026. The metabolite names were searched separately in HMDB and gutMGene as ‘acetate/acetic acid’, ‘propionate/propionic acid’ and ‘butyrate/butyric acid’. When a human gene or protein was explicitly reported as being associated to the corresponding metabolite in the record of the database, candidate interaction was retained. Redundant duplicates were eliminated and protein identifiers were standardized to official symbols for the human genome. The chemical identifiers and canonical SMILES were retrieved and verified from PubChem, and were not deemed to be independent evidence of a validated (15–17). Detailed information on the input numbers principal analytical parameters used throughout the computational workflow is provided in **Supplementary Table S1**.

### 2.2 Protein–Protein Interaction (PPI) network construction

The interactome targeting SCFAs was represented by the creation of a protein-protein interaction (PPI) network generated through the use of STRING (https://string-db.org). The identification of the functional architecture in this network was achieved by clustering the network, that is, assigning nodes to particular clusters based on metabolic, epigenetic, and signalling modules. This process was guided by high connection density within each cluster as well as curated functional annotations. The selected SCFA-related host targets were uploaded to the STRING database v12.5, accessed on 8^th^ April 2026 for protein–protein interaction (PPI) analysis and the organism was limited to Homo sapiens. The minimum required interaction confidence score was 0.400. The interaction sources were protein interactions and co expression network. The PPI network was then imported into Cytoscape v 3.10.4 and visualized and analyzed topologically (18).

### 2.3 Hub gene identification and network centrality analysis

Identification of the most critical regulatory nodes in the network was achieved via topological prioritization in Cytoscape (https://cytoscape.org) v 3.10.4 with the use of the CytoHubba plugin (https://apps.cytoscape.org/apps/cytohubba) v0.1 accessed on 10^th^ April 2026. The significance of each gene was evaluated based on its centrality measures, mainly degree centrality and betweenness. Degree and betweenness centrality were predefined as the principal measures for hub prioritization in the present analysis. Other network-centrality algorithms, including Bottleneck, Eccentricity, and Maximal Clique Centrality (MCC), were not incorporated into the hub-selection procedure. Therefore, the identified hubs represent prioritization according to connectivity and intermediary positioning rather than consensus across multiple centrality algorithms. Genes were ranked according to their centrality scores, such that the ones with high connectivity and control over the SCFA-host network were selected for further analysis. The cytoHubba plugin version v0.1 was used to perform the hub-gene analysis in v 3.10.4. A list of the top 10 genes was chosen to be hub genes based on the pre-determined gene ranking and then subjected to downstream analysis of expression, survival, immune-infiltration and integrated computational analysis (19, 20). Hub-gene prioritization was performed sequentially rather than on the basis of a single network metric. The initially prioritized genes were carried forward for evaluation across complementary analytical layers, including expression profiling, relapse-free survival, gene–metabolite connectivity, and immune-infiltration analyses. Genes showing convergent evidence across these analyses were subsequently selected for more focused genomic, epigenetic, and therapy-response characterization. This integrative prioritization resulted in the selection of STAT3 and HIF1A for focused downstream evaluation.

### 2.4 Clinical expression profiling in breast cancer

Expression profiles of the selected hub genes in breast invasive carcinoma (BRCA) were examined using GEPIA2 (http://gepia2.cancer-pku.cn) accessed on 12^th^ April 2026. These samples were therefore treated as tumor and normal comparison groups and not as patient-matched samples. In order to guarantee homogeneity in the gene expression profiles, the log_2_(TPM + 1) scaling transformation was utilized as the expression measurement index (21).

### 2.5 Survival and prognostic analysis

The Kaplan–Meier Plotter database was used to assess the prognostic value of the 10 hub genes respectively, with relapse-free survival (RFS) as the clinical endpoint. Patients were classified as high or low expression groups based on the expression cutoff values determined from the Kaplan–Meier Plotter analysis based on the Kaplan-Meier Plotter database (https://kmplot.com) accessed on 13^th^ April 2026. The Affymetrix probe sets analyzed were *IL1B* (39402_at), *TLR4* (232068_s_at), *TNF* (207113_s_at), *IL10* (207433_at), *CCL2* (216598_s_at), *PPARG* (208510_s_at), *HIF1A* (200989_at), *IL6* (205207_at), *JUN* (201466_s_at), and *STAT3* (225289_at). The differences in survival between expression groups were assessed with the log-rank test, and the hazard ratios (HRs) and 95% confidence intervals (CIs) were presented. A p-value of < 0.05 was deemed statistically significant. As ten hub genes were tested and without multiple testing correction, the survival analysis was exploratory and the associations observed were interpreted as suggestive rather than causal. Survival differences between the high- and low-expression groups were evaluated using the log-rank test, and hazard ratios (HRs) with 95% confidence intervals (CIs) were reported. A nominal P < 0.05 was considered statistically significant. No multiple-testing correction was applied across the ten evaluated hub genes; therefore, these analyses were considered exploratory and interpreted cautiously (22).

### 2.6 Integrative gene–metabolite network analysis

In order to explore the convergence of functions between the signaling pathway and metabolic pathway, a hybrid network combining gene-metabolite interactions was built with the help of the Network Explorer module within the MetaboAnalyst 6.0 (https://www.metaboanalyst.ca) accessed on 15^th^ April 2026. The hub metabolic molecules were detected from the network topology according to their higher values of degree and betweenness centrality, which were used to build the connection map between the host metabolism and the signaling pathways (23).

### 2.7 Immune infiltration and tumor microenvironment analysis

The connection between signaling through SCFA receptors and the response from the host immune system was assessed based on the correlation of hub genes with tumor-infiltrating CD8+ T cells using the TIMER2.0 database (https://compbio.cn/timer2) accessed on 17^th^ April 2026. In order to assess a comprehensive immune context for all the subtypes of breast cancer, different computational approaches like CIBERSORT, QUANTISEQ, and XCELL were used to determine their infiltration levels. Associations between hub-gene expression and computationally estimated immune-cell abundance were assessed using the correlation statistics provided by TIMER2.0. A nominal P < 0.05 was used to identify nominally significant correlations; because no multiple-testing correction was applied, these associations were considered exploratory. Where multiple comparisons were not adjusted, the results were treated as exploratory. (24).

### 2.8 Promoter methylation analysis

The promoter methylation status of specific genes responsive to SCFAs was assessed using the online tool, UALCAN (https://ualcan.path.uab.edu) accessed on 20^th^ April 2026. The TCGA Breast Invasive Carcinoma dataset was chosen, and the comparison of promoter methylation beta values was carried out in both normal breast tissue and breast tumors. Promoter methylation analysis of *HIF1A* and *STAT3* was performed to assess whether any epigenetic changes might be responsible for the dysregulation of expression of these genes in breast cancer (25).

### 2.9 Therapy response prediction

To assess the potential utility of hub genes responsive to the selected SCFAs in predicting responses to treatments in breast cancer, we used the ROC Plotter server (https://rocplot.com) accessed on 23^rd^ April 2026. *HIF1A* and *STAT3* were selected to evaluate their association with pathological treatment response in terms of their diagnostic value concerning the pathological response to the treatment of breast cancer using endocrine, anti-HER2, chemotherapy, or combination therapy. Gene expressions were compared for treatment responders and non-responders, and their diagnostic utility was analysed via ROC curves. The parameters utilized for this purpose include area under the curve, ROC P-value, Mann-Whitney U Test P-value, and fold change. Predictive performance was assessed using the area under the receiver operating characteristic curve (AUC), with 95% confidence intervals reported where available. Statistical significance was evaluated using the P-values generated by ROC Plotter, with P < 0.05 considered nominally significant. AUC magnitude, rather than statistical significance alone, was used to interpret discriminatory performance (26).

### 2.10 Genomic alteration and mutational landscape analysis

In order to analyse the genome stability of the discovered hub genes, genetic profiling was done through the cBioPortal for Cancer Genomics (https://www.cbioportal.org) accessed on 25^th^ April 20226. This involved an evaluation of various frequencies of alterations, where the genomic events were classified into amplifications, deep deletions, and missense mutations. The genetic profiling was done using the TCGA Breast Invasive Carcinoma (Pan-Cancer Atlas) cohort, giving a complete picture of how these metabolic and transcriptional biomarkers are modified in breast cancer cases (27).

### 2.11 Drug–gene interaction and therapeutic repurposing

For the assessment of the translational relevance of the identified hub genes, drug target identification was performed using the Drug-Gene Interaction Database (DGIdb) (https://dgidb.org) accessed on 27^th^ April 2026. This tool served as an instrument for the identification of a diverse range of small molecules and clinically approved inhibitors that physically interact with or functionally affect the SCFA-responsive network. Translational prioritization of targets was done by selecting compounds that have a strong repurposing potential, considering their connectivity and potency as inhibitors of the central metabolic and signaling hubs within the breast cancer network, like *STAT3*, *TNF*, and *IL6*. The identified interactions represent previously reported or database-curated drug–gene relationships and were not interpreted as evidence of therapeutic efficacy, target engagement, selectivity, or clinical effectiveness in breast cancer. Candidate compounds were subsequently classified according to their reported interaction/evidence category and clinical relevance, where such information was available from the database. (28).

## 3 Results

### 3.1 Targeted mapping of SCFA–protein interactions

The analysis of the interaction landscape of SCFAs and human host targets revealed a complex yet coordinated network regulating multiple signaling pathways. The most significant interactome was found for butyrate, featuring its ability to potently inhibit multiple histone deacetylases (HDAC1, 2, 3, 8, and 11). causally active. Consistent trends were discovered in the signaling pathways of acetate and propionate, with both being capable of activating FFAR2/3 receptors and barrier proteins such as OCLN and TJP1. All three SCFAs had the ability to induce ALPI and regulate the activity of the ANGPTL4 metabolic rheostat. These findings demonstrate a conserved mechanism by which microbiota-derived metabolites affect host metabolism and signaling processes. The results suggest that SCFAs are able to modulate multiple levels of interactions in human host cells, from sensing via FFAR receptors to epigenetics and metabolism.

### 3.2 Network Topology and Functional Organization of the SCFA–Host Protein Interaction Network

The analysis of the topological characteristics of the SCFA-target interactome was performed based on the PPI network generated via the STRING database. As shown in the topographical representation, this protein network has high density and connectivity since the number of interactions between nodes was much higher than random probability, indicating that this set of proteins forms a functionally interconnected network rather than a set of isolated proteins. As shown in the map, there were three separate modules: a metabolic module, mainly consisting of ACSS2, ACLY, and FASN enzymes; an epigenetic regulatory module including HDAC family proteins and SIRT1; and the largest was the signaling/immunomodulatory module composed of FFAR2/3 and several interleukin molecules. Notably, the existence of thick edges connecting the HDAC module and the metabolic module Connections were also observed between proteins assigned to the epigenetic and metabolic modules within the STRING-derived PPI network. **Figure 1** below represents the *Network topology analysis* of the SCFA–host target PPI network.

**Figure 1:**
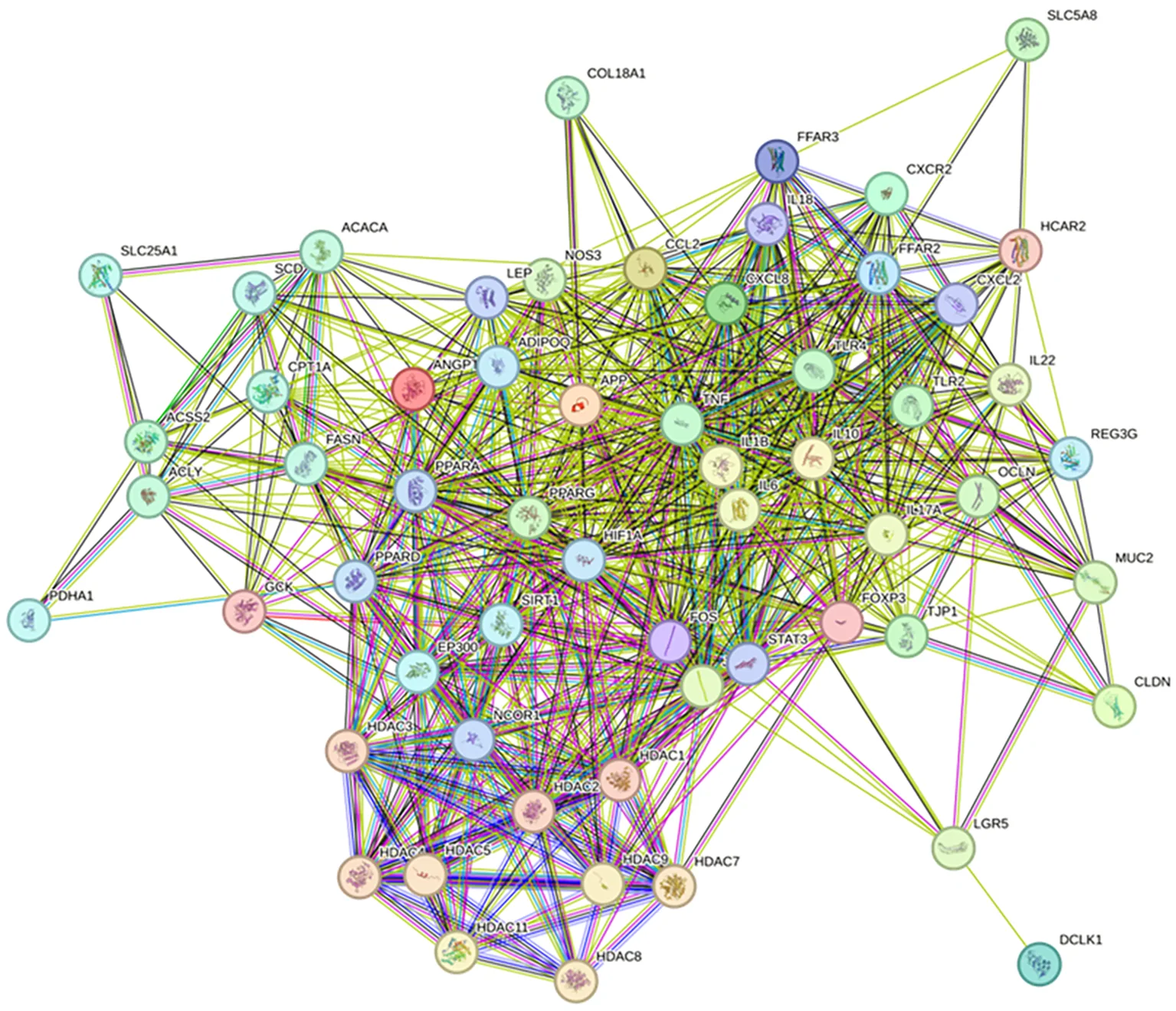
Network topology analysis of the SCFA–host target PPI network. The network, visualized via the STRING database, illustrates the complex interconnectivity between gut-metabolite-responsive genes. Nodes represent individual proteins, while edges represent the strength and nature of the interactions based on experimental evidence, co-expression, and curated databases. Distinct functional modules are visible, including the HDAC epigenetic cluster, the fatty acid and Acetyl-CoA metabolic cluster (highlighted by the red dotted circle), and the G-protein coupled receptor (GPCR) signalling axis.

### 3.3 Identification and prioritization of HUB genes

The identification of the most influential nodes in the SCFA–host interactome was done using a cytoHubba analysis and the degree centrality algorithm. In terms of priority, the top 10 genes were ranked in relation to their importance and connectivity within the network in descending order of importance, symbolized by a color gradient from red to yellow. As a result, *IL6* and *IL1B* were identified as the two most highly connected hub genes, owing to their maximum connectivity levels, with *TNF* and *CCL2* coming close behind them. Notably, the metabolic and transcriptional regulator genes like *PPARG*, *HIF1A*, and *STAT3* appeared as important biomarkers for enabling crosstalk between inflammation signaling and cell regulation. Based on degree-centrality ranking, IL6 and IL1B showed the highest connectivity, followed by other prioritized nodes including TNF, CCL2, PPARG, HIF1A, STAT3, JUN, TLR4, and IL10. These ten genes were retained for subsequent analyses. **Figure 2** below represents the constructed HUB Gene Network representing the top 10 priority genes in the predicted protein interaction network.

**Figure 2:**
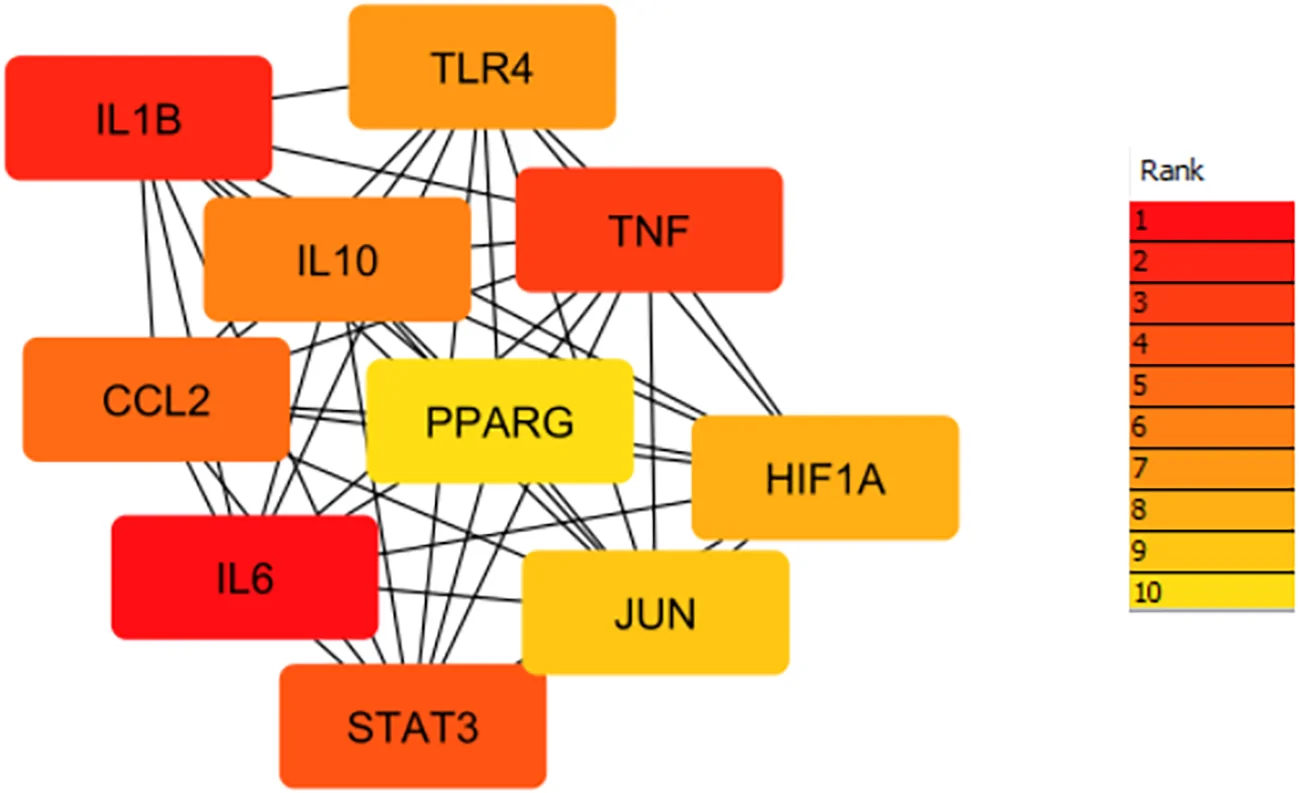
Identification of top hub genes within the SCFA-host interaction network. The network was analyzed using the CytoHubba plugin in Cytoscape to rank proteins based on their degree centrality. The color-coded nodes represent the top 10 prioritized targets, where bright red signifies the highest degree of connectivity (e.g., *IL6, IL1B*) and yellow indicates the lower-tier hub genes (e.g., *PPARG, JUN*). The lines (edges) represent the physical and functional interactions connecting these central regulatory nodes.

### 3.4 Clinical filtering and expression profiling in breast cancer

In order to determine the significance of the prioritized SCFA-responsive hub genes in terms of clinical relevance, the expression patterns of the selected target genes were assessed in TCGA-BRCA patients using the GEPIA2 web server. From the differential expression analysis, the analysis showed distinct expression patterns among the selected hub genes in breast cancer tissues for the selected hub genes. Some of the main regulatory targets that are up-regulated in breast cancer include *STAT3*, *JUN*, *HIF1A*, and *CCL2*, where the median expression value attained the highest expression in the blue-scale heat map plot. On the other hand, despite some of the target genes (*IL1B*, *TLR4*, and *PPARG*) being moderately expressed in the tissue, cytokines such as *TNF*, *IL10*, and *IL6* exhibited low basal expression in the tumor bulk samples. **Figure 3** below represents the Expression profiling of SCFA-target hub genes in the TCGA Breast Invasive Carcinoma (BRCA) cohort.

**Figure 3:**
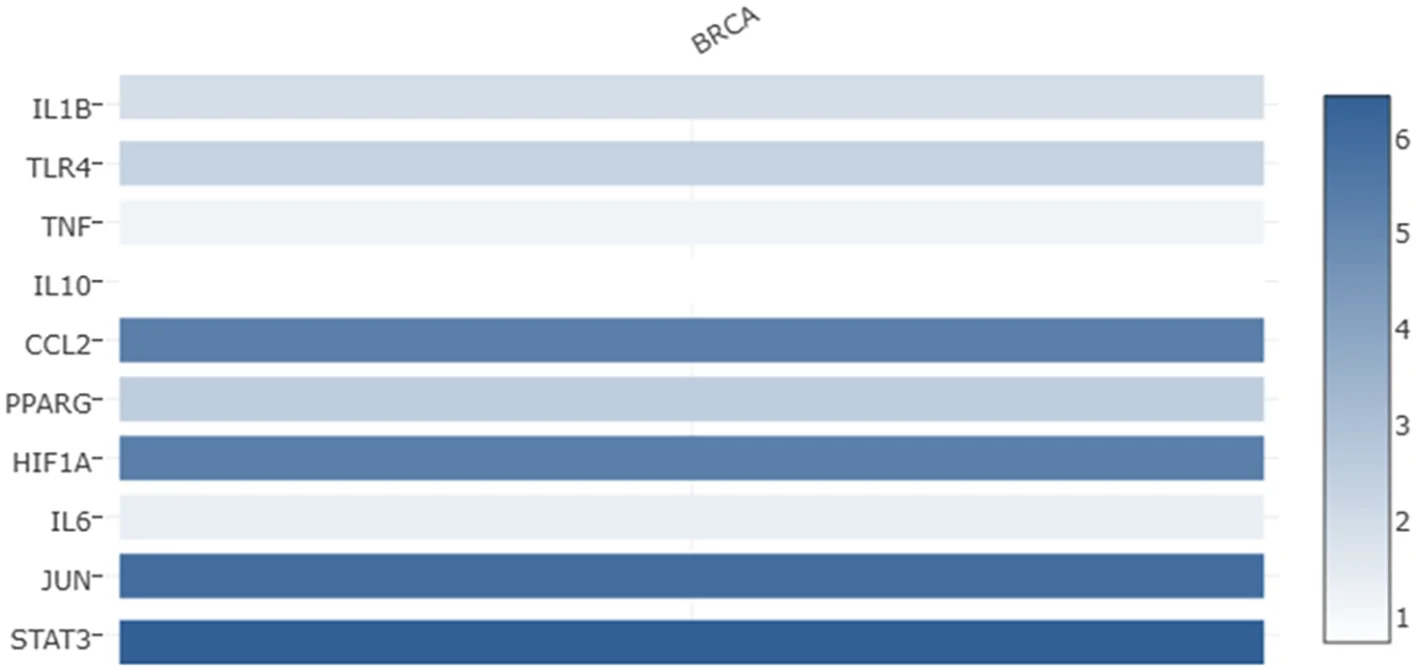
Expression profiles of selected SCFA-associated hub genes in breast invasive carcinoma (BRCA). The plot shows relative expression levels of the selected genes. The visualization is descriptive and does not represent a statistical differential-expression analysis.

### 3.5 Prognostic significance via survival analysis

Relevance to the prognosis of the selected hub genes has been established by assessing the influence of the prioritized genes on RFS in breast cancer patients via the Kaplan-Meier plotter. Several hub genes showed statistically significant associations with RFS as they showed statistically significant correlations. Thus, increased expression of *HIF1A* (HR = 1.39, p = 1.6e-10) and *STAT3* (HR = 1.19, p = 0.022) is associated with poor prognosis in breast cancer, which indicates that the expression of these genes is associated with disease outcome; however, these associations do not establish a causal role in disease progression. At the same time, high levels of inflammatory and signaling molecules like *IL1B* (HR = 0.78, p = 1.2e-06), *TNF* (HR = 0.8, p = 9.5e-06), *TLR4* (HR = 0.75, p = 0.00014), *PPARG* (HR = 0.84, p = 0.00082), and *IL10* (HR = 0.84, p = 0.00077) were associated with improved RFS. The basal expression levels of *IL6* in the bulk tumour expression analysis were relatively low, but elevated expression of *IL6* in the overall cohort was weakly correlated with RFS (HR = 0.90, p = 0.046). The results are not directly contradictory, as the two analyses (expression and survival) compare different aspects: the expression analysis characterizes the overall abundance of a transcript, while the survival analysis assesses the relative expression differences between patients. Due to the low effect size and close to no statistical significance, the association of *IL6* should be considered with caution and is not indicative of a protective effect. **Figure 4** below represents the Kaplan-Meier survival curves illustrating the prognostic value of SCFA-target hub genes in breast cancer.

**Figure 4:**
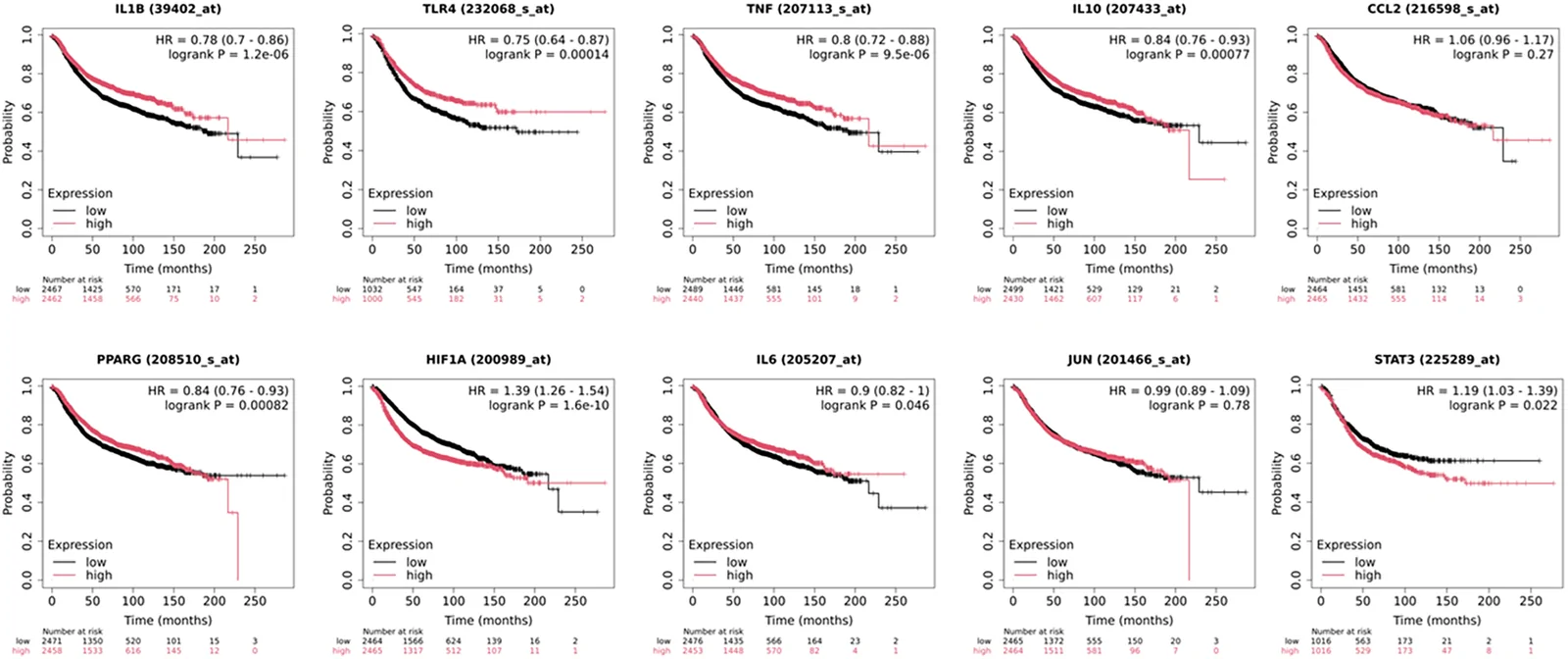
Kaplan–Meier analysis of relapse-free survival (RFS) according to expression of ten SCFA-associated hub genes in breast cancer. Patients were stratified into high- (red) and low-expression (black) groups. Kaplan–Meier curves are shown for *IL1B*, *TLR4*, *TNF*, *IL10*, *CCL2*, *PPARG*, *HIF1A*, *IL6*, *JUN*, and *STAT3*. Hazard ratios (HRs) with 95% confidence intervals and log-rank p-values are displayed for each analysis. *HIF1A* and *STAT3* expression was associated with shorter RFS, whereas *IL1B*, *TLR4*, *TNF*, *IL10*, *PPARG*, and *IL6* showed associations with longer RFS. *CCL2* and *JUN* were not significantly associated with RFS.

### 3.6 Multi-omic pathway and functional integration

Integration of SCFA-responsive gene networks with the metabolic landscape led to the identification of a highly intricate regulatory network between inflammation and metabolism. Topological characterization of the network formed between genes and metabolites pinpointed *IL6* and *PPARG* as the two bridging molecules showing the highest degree and betweenness scores. Network topology identified IL6 and PPARG as highly connected bridging genes based on degree and betweenness centrality. STAT3, JUN, and HIF1A were also represented among the connected nodes. Therefore, this association was considered exploratory and requires independent validation. Thus, its presence in the network indicates a possible cross-talk between host signaling pathways involved in SCFA and other metabolic pathways, to be confirmed by further mechanistic validation. **Figure 5** below represents the Integrative gene–metabolite interaction network of SCFA-responsive targets.

**Figure 5:**
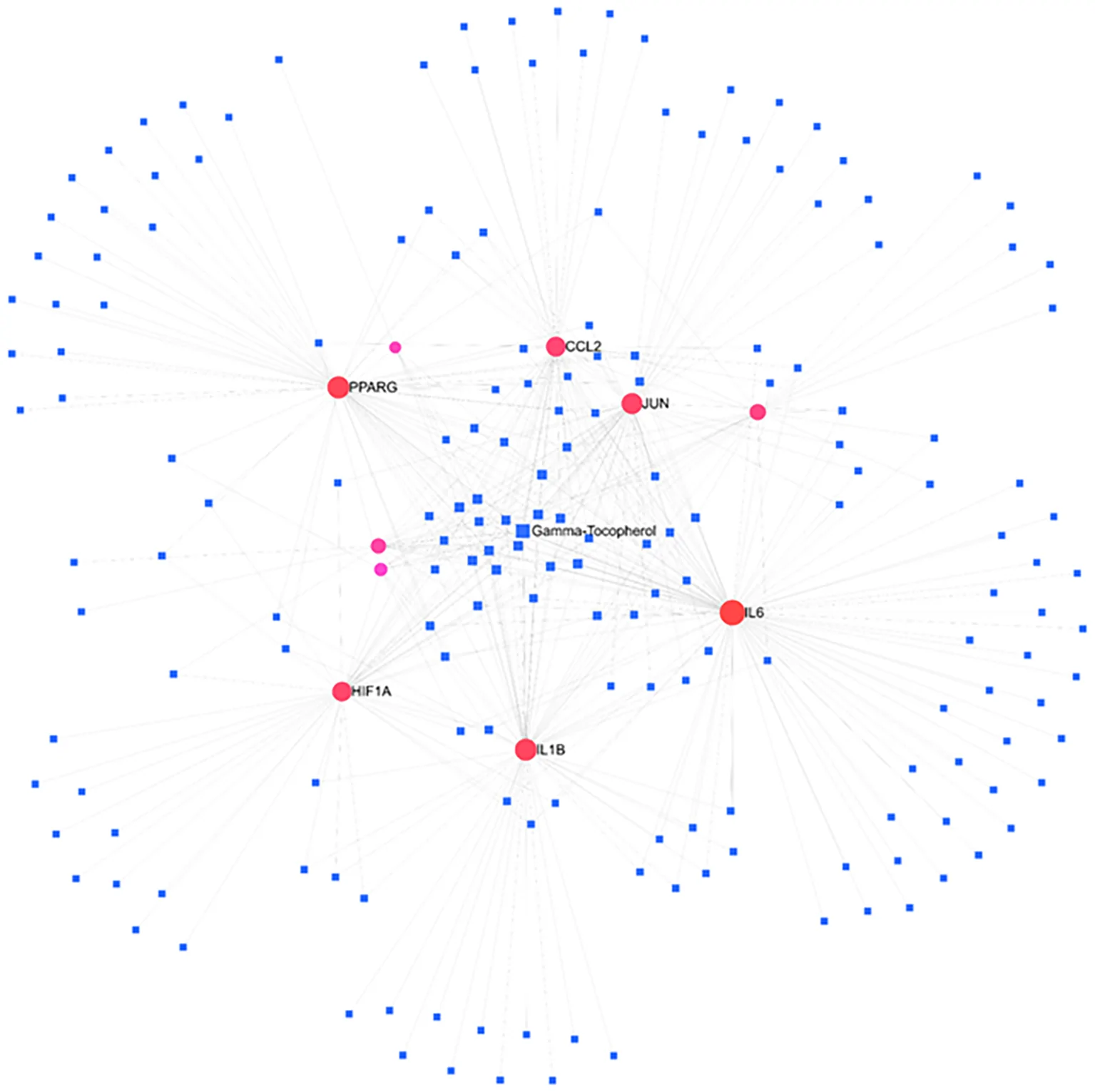
Integrative gene–metabolite interaction network of SCFA-responsive targets. The network illustrates the topological connectivity between prioritized hub genes (red nodes) and their associated metabolites (blue squares). Node size and arrangement are determined by centrality measures, highlighting *IL6*, *PPARG*, and *IL1B* as the primary regulatory anchors. Gamma-.

### 3.7 Tumor Microenvironment (TME) and immune infiltration correlation

Immune infiltration analysis showed that the expression of some SCFA-associated hub genes was positively correlated with the EI of CD8+ T-cells in breast cancer subtypes. Results from the heatmap presented in **Figure 6** indicated that the directions and magnitudes of these associations were different for genes, BRCA subtypes and immune-deconvolution algorithms. A positive trend was seen in all the three estimation methods (CIBERSORT, QUANTISEQ and XCELL) and statistically significant correlation was found at p value ≤ 0.05. Not all of the associations were equally strong and impactful, however. The results suggest a correlation between the genes within the network and the immune cell composition of the breast tumor microenvironment, but do not prove that the activation of CD8+ T cells is directly caused by the SCFA. TIMER2.0 analysis identified correlations between the expression of selected SCFA-associated hub genes and computationally estimated CD8+ T-cell abundance. **Figure 6** presents these associations across multiple cancer types and therefore represents a pan-cancer overview. The direction and magnitude of the correlations varied across tumor types and immune-deconvolution algorithms. Accordingly, these associations should not be interpreted as evidence that SCFAs directly increase, recruit, or activate CD8+ T cells.

**Figure 6:**
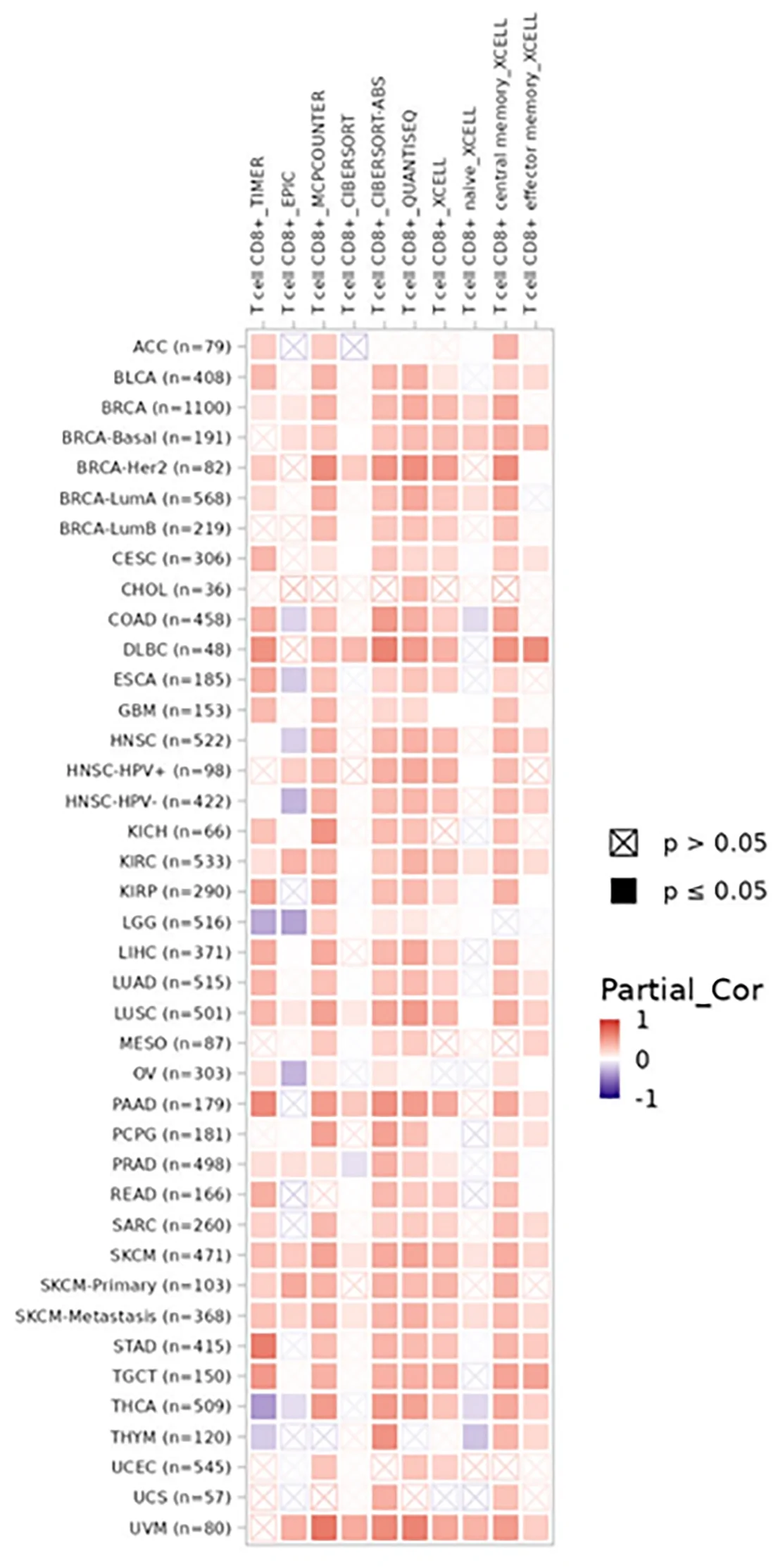
Pan-cancer association between SCFA-associated hub-gene expression and estimated CD8+ T-cell infiltration. The heatmap presents partial correlation coefficients across multiple human cancer types using TIMER2.0 immune-deconvolution algorithms. Red and blue indicate positive and negative correlations, respectively. Solid squares indicate nominally significant associations (P < 0.05), whereas crossed squares indicate P ≥ 0.05.

### 3.8 Genetic alteration and mutation landscape of metabolic hubs

Genomic stability and mutation profiles for the hub genes identified above were assessed in the TCGA-BRCA Pan-Cancer Atlas to understand their evolutionary stability and degree of alteration in clinical cases. It was found that there is a significant rate of genetic alteration within the hub gene set, where individual hubs showed a range of alterations from 4 to 15 percent. Genomic alteration frequencies varied across the analyzed breast cancer cohorts and among the selected hub genes, with mutations and copy-number alterations contributing to the observed alteration profiles. These genomic findings were analyzed independently of the transcriptomic results. Therefore, although genomic alterations and expression differences may coexist for some genes, the present analysis does not demonstrate that amplification or mutation is responsible for the observed expression pattern. Meanwhile, inflammatory genes, such as *IL1B* and *TNF*, showed lower but distinctive genetic landscapes of alteration characterized by occasional deep deletions and structural variations. Therefore, despite some SCFA targets remaining genetically stable, there are certain biomarkers related to metabolic processes or transcription in cancer cells undergoing frequent genomic amplification. The summary of alteration frequencies per gene is given in **Figure 7** below.

**Figure 7:**
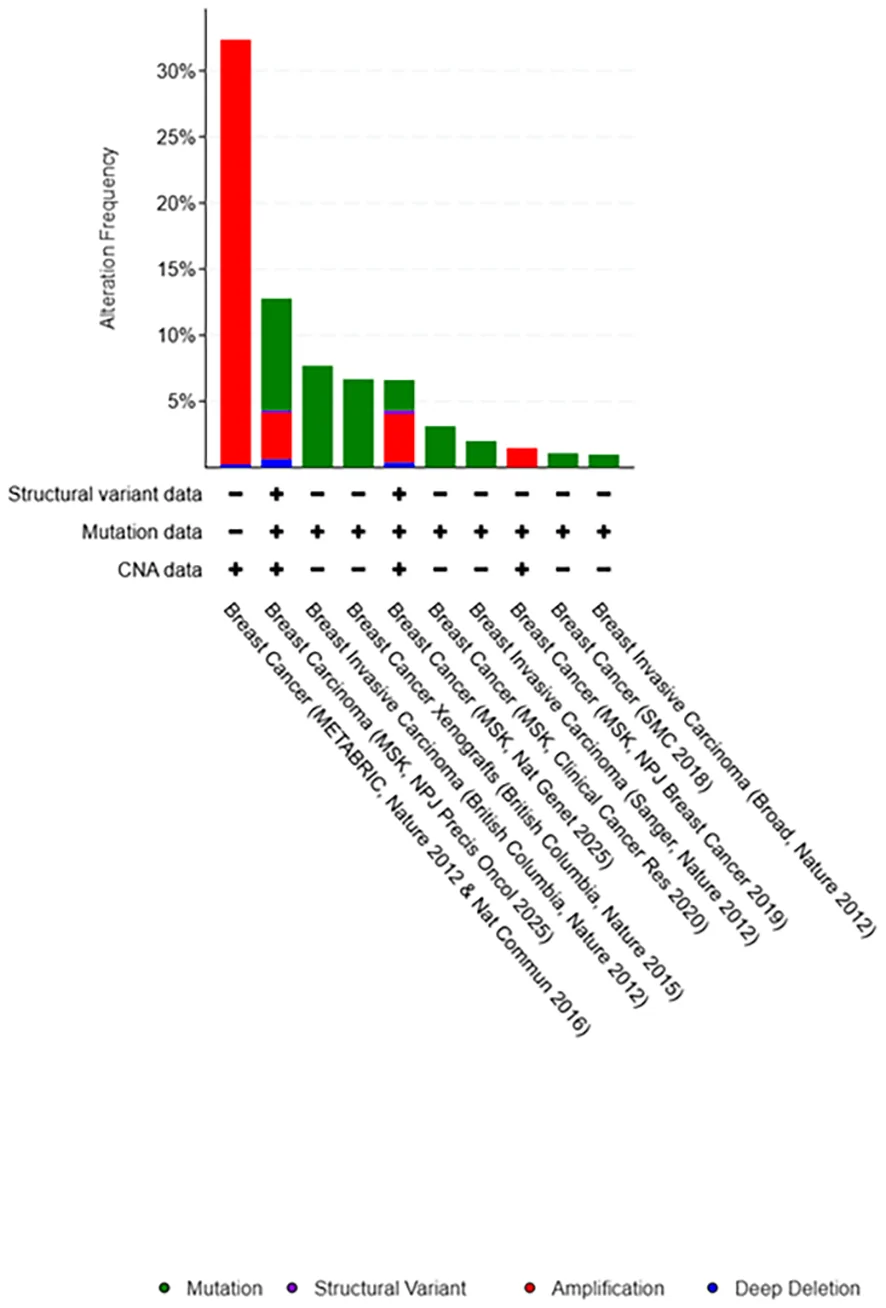
Genomic alteration profiles of the selected SCFA-associated hub genes across breast cancer cohorts available in cBioPortal. The image summarizes the frequency and distribution of genomic alterations, including mutations and copy-number alterations, across the included studies. Alteration profiles are descriptive and do not establish a causal relationship with gene expression.

### 3.9 Promoter methylation analysis

Methylation status analyses were conducted to assess whether promoter methylation may contribute to the dysregulation of certain selected SCFA-dependent key genes in breast cancers. According to the TCGA-BRCA dataset, *HIF1A* demonstrated slightly higher promoter methylation, in addition to an increased spread of beta values, in comparison with normal breast tissues. The results could mean that *HIF1A* could be subject to various degrees of epigenetic control during the process of tumor growth in breast tissue. Demethylation of HIF1A and STAT3 in primary breast tumors compared with normal breast tissues were found to be only modestly descriptive by promoter methylation analysis. Due to the lack of sufficient statistical evidence and estimates of effect sizes from the present analysis, no significant difference is claimed. These observations are therefore descriptive and would not prove HIF1A or STAT3 to be promoter methylation regulated. The **Figure 8** below represents the promoter methylation profiles of HIF1A and STAT3 in TCGA-BRCA samples.

**Figure 8:**
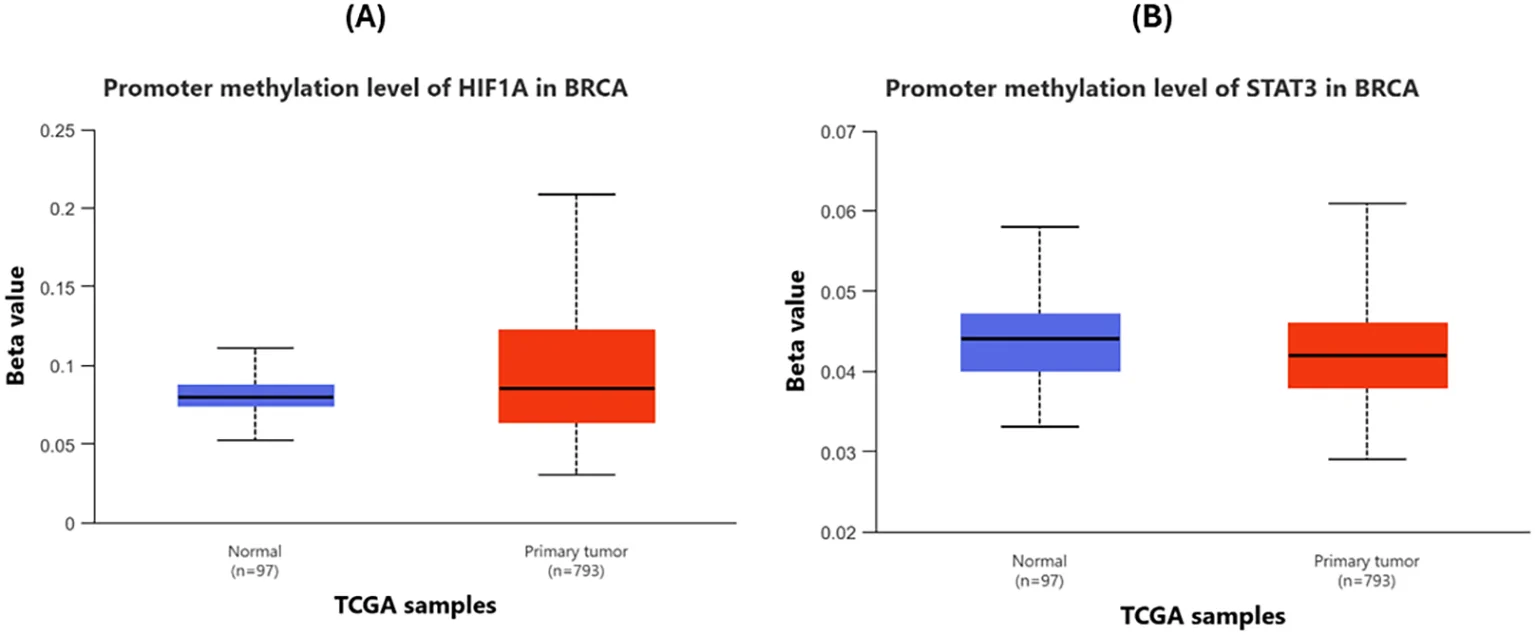
Promoter methylation profiles of HIF1A and STAT3 in TCGA-BRCA samples. **(A)** HIF1A and **(B)** STAT3 promoter methylation levels in normal breast tissue and primary breast tumors. The observed differences are presented descriptively, and no significant difference is claimed based on the present analysis.

### 3.10 Therapy response prediction

The predictive association of the chosen SCFA-responder hub genes was further studied using ROC Plotter to see how well they predicted pathological response to various treatments for breast cancer, such as endocrine therapy, anti-HER2 therapy, and chemotherapy. Among the analyzed genes, HIF1A demonstrated the highest predictive power with respect to distinguishing responders from non-responders. Indeed, its expression level was higher among responder patients – its mean expression value was 4733, which is 3977 for non-responders, showing a difference of 1.2. ROC analysis showed that HIF1A achieved an AUC of 0.599 (p = 2.8 × 10^−12^), indicating only modest discriminatory performance despite the statistically significant association. Therefore, the statistical significance should not be interpreted as evidence of clinically useful predictive accuracy. In contrast, STAT3 showed an AUC of 0.504 (p = 0.45), indicating essentially no discriminatory ability for treatment response. These findings suggest that neither marker demonstrated sufficient standalone predictive performance to support clinical application based on the present analysis. The **Figure 9** below represents the therapy response prediction of HIF1A and STAT3 in breast cancer using ROC Plotter.

**Figure 9:**
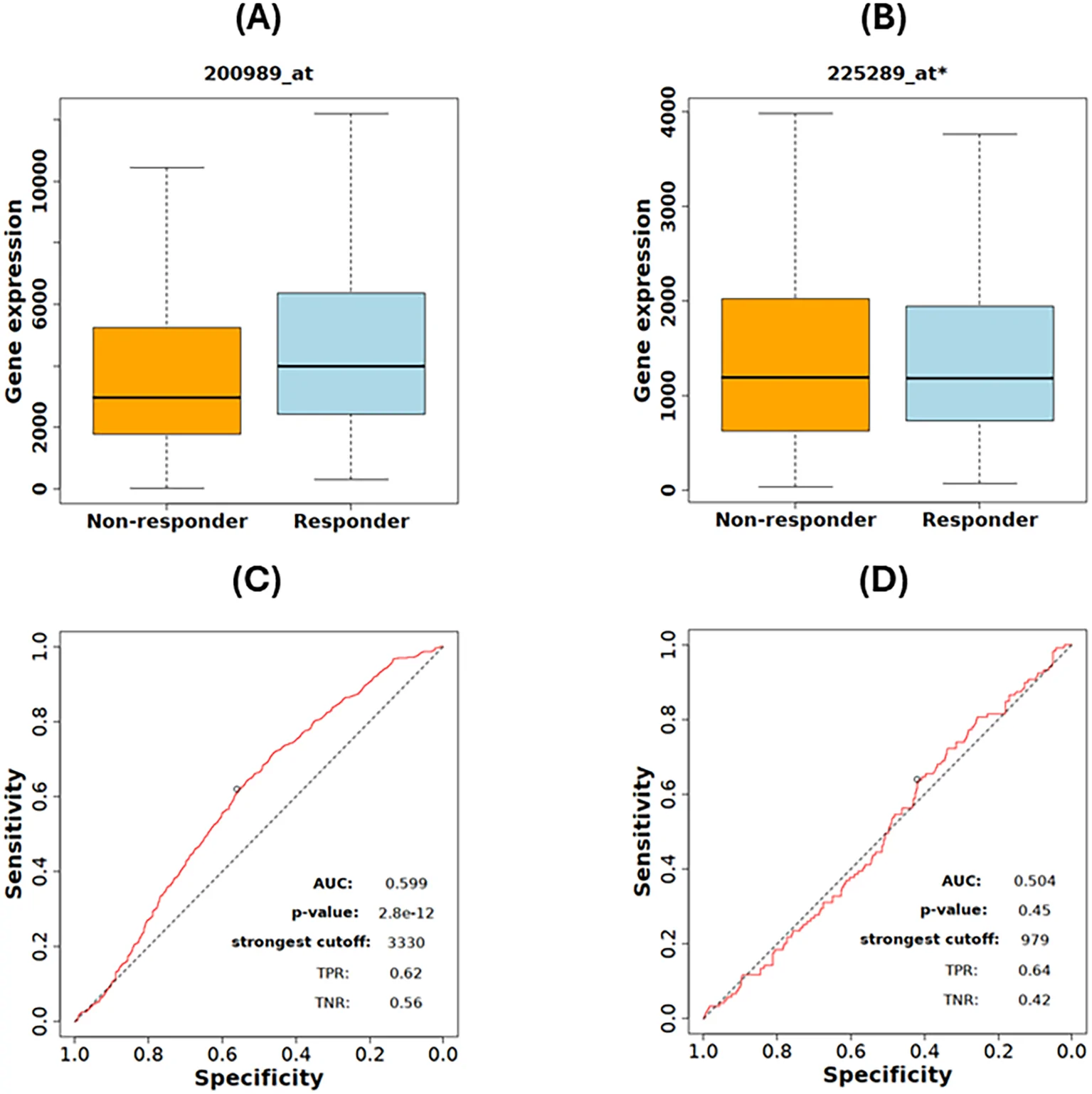
Therapy response prediction of HIF1A and STAT3 in breast cancer using ROC plotter. **(A)** HIF1A (probe 200989_at) expression in responders and non-responders and the corresponding ROC curve (AUC = 0.599), indicating modest discriminatory performance. **(B)** STAT3 (probe 225289_at) expression in responders and non-responders and the corresponding ROC curve (AUC = 0.504), indicating essentially no discriminatory performance. Box plots show gene-expression distributions according to treatment response, while ROC curves show the ability of each marker to distinguish responders from non-responders.

### 3.11 Literature-supported drug–gene associations and therapeutic repurposing potential

As a result, an analysis of potential drug–gene interactions was performed to determine which pharmacological substances may affect the SCFA-responsive hub gene network and to provide further validation of their translational potential. Several promising candidates were detected, revealing an extensive pharmacological landscape. Particularly, among those genes, STAT3 showed interactions with multiple pharmacological agents, with numerous agents available that could affect its function. For example, the gene was found to interact with anti-inflammatory drugs Celecoxib, Pyrimethamine, and the natural substance Cucurbitacin I. In addition, TNF and IL6 genes showed substantial connectivity with anti-inflammatory drugs and biologicals, such as Infliximab, Adalimumab, and Tocilizumab. **Figure 10** below represents the DGIdb-curated drug–gene associations and therapeutic repurposing potential.

**Figure 10:**
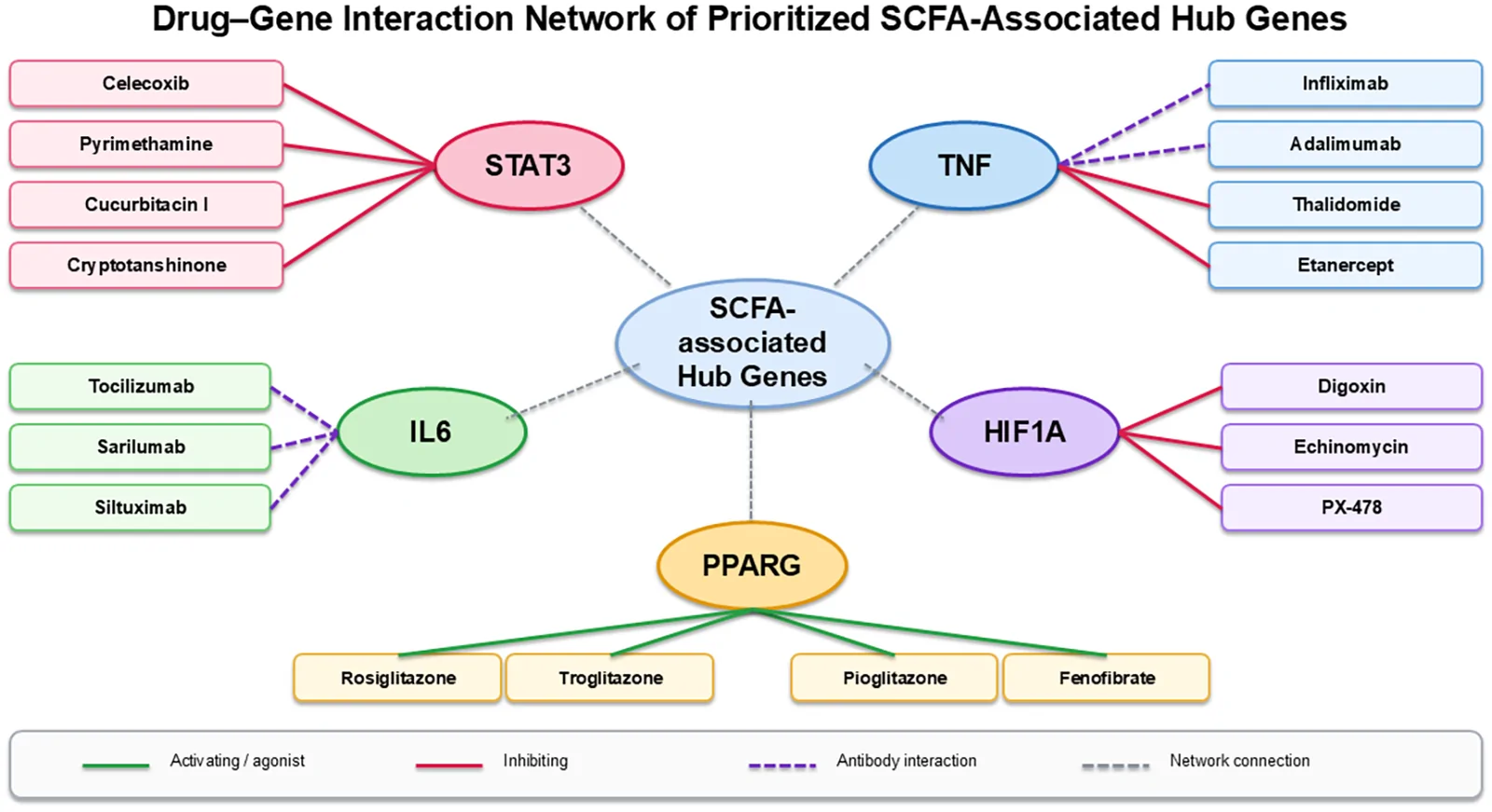
Drug–gene interaction network of prioritized SCFA-associated hub genes. The network represents DGIdb-curated associations between prioritized hub genes and their corresponding drugs or compounds. Gene and drug nodes are connected by edges representing reported drug–gene relationships, with interaction types (e.g., inhibitor, agonist, antagonist, or antibody) indicated where available. These associations represent database-curated evidence and do not establish therapeutic efficacy in breast cancer.

PPARG was associated with several metabolic modulators, such as Rosiglitazone and Troglitazone. Reprogramming. **Table 1** below represents the list of interacting drugs with the identified HUB Genes.

**Table 1:** Prioritized hub genes and potential therapeutic repurposing agents.

Hub gene	Drug/small molecule	Interaction type	Evidence level/source
STAT3	Celecoxib	Inhibitor	DGIdb-curated association
STAT3	Pyrimethamine	Inhibitor	DGIdb-curated association
STAT3	Cucurbitacin I	Inhibitor	DGIdb-curated association
STAT3	Cryptotanshinone	Inhibitor	DGIdb-curated association
TNF	Infliximab	Antibody/inhibitor	DGIdb-curated association
TNF	Adalimumab	Antibody/inhibitor	DGIdb-curated association
TNF	Thalidomide	Inhibitor	DGIdb-curated association
TNF	Etanercept	Inhibitor	DGIdb-curated association
IL6	Tocilizumab	Antibody/pathway inhibitor	DGIdb-curated association
IL6	Sarilumab	Antibody/pathway inhibitor	DGIdb-curated association
IL6	Siltuximab	Antibody/inhibitor	DGIdb-curated association
PPARG	Rosiglitazone	Agonist	DGIdb-curated association
PPARG	Troglitazone	Agonist	DGIdb-curated association
PPARG	Pioglitazone	Agonist	DGIdb-curated association
PPARG	Fenofibrate	Agonist/modulator	DGIdb-curated association
HIF1A	Digoxin	Inhibitor	DGIdb-curated association
HIF1A	Echinomycin	Inhibitor	DGIdb-curated association
HIF1A	PX-478	Inhibitor	DGIdb-curated association

## 4 Discussion

Integrated computational analysis of the present study has revealed a highly orchestrated network, where gut-derived SCFAs control the epigenetic and metabolic landscapes of the host. The present study points out to a highly significant role played by *STAT3*, *HIF1A*, and *PPARG*, which serve as key metabolic regulators overexpressed in clinical breast cancer specimens (29, 30). Overexpression and genetic aberrations of transcription factors, such as *HIF1A* and *STAT3*, have been identified to contribute to poor relapse-free survival in patients with breast cancer. On the other hand, certain immune-regulating SCFA target genes have shown an association with improved patient outcomes and CD8+ T cell infiltration (31–33). The results imply that the relationship between SCFAs generated by microbiota and breast cancer involves the concept of two-way regulation, combining epigenetic changes with metabolic shifts (34). The identification of *STAT3* and *HIF1A* as central network nodes associated with unfavorable prognosis suggests a potential convergence between SCFA-associated signaling, inflammatory regulation, and hypoxia-related pathways in breast cancer. The observed positive associations between several hub genes and CD8+ T-cell infiltration further suggest a possible relationship between SCFA-associated signaling networks and the breast cancer immune microenvironment; however, these correlations do not establish direct SCFA-mediated immune modulation (35). The involvement of the ALPI/ANGPTL4 axis and the pharmacological connectivity of *STAT3* identified in our analysis provide candidate pathways for investigating potential metabolic and signaling interventions (35, 36).

The interaction between the gut microbiome and distal cancers has become an important pathway in the field of oncology, especially within the framework of the gut-breast axis. The role of SCFAs, such as acetate, propionate, and butyrate, which are metabolites produced by the gut microbiota, in the communication between microbes and host cells, including epigenetic mechanisms, has been recognized (9, 37). Although the systemic effect of these metabolites is extensively documented, the role of these metabolites in the metabolic and immunological reprogramming of breast cancer has remained understudied. Using computational data and clinical expressions, this paper examines the role of SCFAs not only as metabolic products but also as regulators in the breast cancer environment (38, 39).

Methylation studies of promoters have further confirmed the epigenetic aspect of the regulation of SCFA-responsive hub genes; methylation variance was found to be higher in the case of *HIF1A* in tumor samples, while in the case of *STAT3*, its methylation variance was lower, implying individualized epigenetic modifications in breast cancers. Although *HIF1A* showed a statistically significant association with treatment response, its AUC of 0.599 indicates limited discriminatory performance, and therefore its potential predictive relevance requires independent validation. *STAT3* showed no meaningful discriminatory performance for treatment response (AUC = 0.504) (26).

The three SCFAs studied in this study are not all the same with respect to the evidence that connects them to the prioritized hub genes. The STAT3 pathway to signaling has been specifically supported by experimental results with propionate, since sodium propionate has been shown to inhibit JAK2/STAT3 in breast cancer models (40). Butyrate is also documented in breast cancer to inhibit viability, migration and invasion of tumor cells, to modulate the expression of TLR4, which are associated with inflammatory and regulatory elements of the identified network, in line with its association with these elements (41). Acetate has a higher representation with aspects related to metabolic and immune regulation, as shown in experimental breast cancer models, in which acetate availability and ACSS2-dependent acetate metabolism can modulate antitumor T-cell function. In the case of direct regulation, though, the link between HIF1A and SCFAs in breast cancer is less clear than propionate–STAT3. Hence, the connections between HIF1A and other hubs should be considered as suggesting hypotheses and not evidence of direct regulation by a specific SCFA (42).

From a mechanistic perspective, the simultaneous activation of *STAT3* and *HIF1A* in the SCFA-responsive network could represent parallel pathways of SCFA signaling and not be a linear pathway. SCFAs can modulate the activity of GPCRs, including FFAR2 and FFAR3, as well as inhibit histone deacetylases (HDACs) (43). The effects on *STAT3* and *HIF1A* induced by these receptors and epigenetics could impact inflammatory and metabolic signaling pathways indirectly. In addition, *STAT3* and *HIF1A* could be considered as interconnected regulatory nodes, as inflammatory signaling and hypoxic responses may converge in the tumor microenvironment. The present network analysis shows association rather than direct regulation by SCFA of either NF-kB or Nrf2, and these mechanistic relationships are thus hypotheses that need to be tested with experiments (44).

It is also important to note that the relationship between the gut–breast axis should be taken with a grain of salt, as direct evidence linking the circulation of microbial metabolites to molecular alterations in human breast tumors is still scant. The concentrations of SCFAs in breast tumors and the exposure to SCFAs in the present study were not measured; therefore, the associations from transcriptomic and immune-infiltration datasets and survival data with tumor phenotypes cannot be used to conclude that gut-derived SCFAs led to the observed tumor phenotypes (45). The value of this study is in the combination of the host targets associated with SCFAs with breast cancer expression, survival, immune, epigenetic, genomic, and drug-gene data in a unified analytic schema, rather than in their ability to establish a causal gut–breast mechanism. This method focuses on *STAT3*, *HIF1A*, *IL6*, and related nodes as potential candidates for further mechanistic studies (46).

While all efforts have been made to incorporate computational integration into this study, there are still some limitations that must be addressed. Firstly, the outcomes of this study rely heavily on in silico analysis and data from public databases; thus, *in vitro* and *in vivo* experiments need to be conducted to establish the causal relationship between SCFA pathways and their functions. Secondly, while the TCGA-BRCA database was employed for this experiment, the concentration of SCFA at a systemic level could not be measured, and therefore, the concentration and flux of SCFA locally within the tumor were estimated using previous publications. Additionally, while there were numerous computational algorithms used to calculate immune cell infiltration, their accuracy is still speculative and must be confirmed using flow cytometry or immunohistochemistry techniques. Hub-gene prioritization was based primarily on degree and betweenness centrality; therefore, future network analyses incorporating complementary measures such as Bottleneck, Eccentricity, and MCC may further assess the stability of the identified hub rankings.

In future studies, more emphasis should be placed on the experimental validation of the results generated in silico by conducting *in vitro* and *in vivo* experiments. Specifically, the goal should be to clarify the mechanism by which SCFAs influence *STAT3* and *HIF1A*. It will also be essential to carry out clinical trials linking gut microbiota composition and levels of SCFAs in the body with the stage of breast cancer and patient outcomes. This will enable researchers to confirm SCFAs as effective biomarkers for breast cancer detection and treatment. Moreover, it would be prudent to explore the therapeutic potential of combining SCFA therapies like diets rich in fiber and postbiotics with repurposing *STAT3* and *TNF* inhibitors, as established in this study.

## 5 Conclusions

In this integrative computational study, three core host signaling molecules, STAT3, HIF1A and IL6, were identified as hubs connecting SCFA-associated signaling pathways to molecular and clinical traits of breast cancer. The correlation of selected hub genes with infiltration of CD8+ T cells indicated possible links to the tumor immune microenvironment, while STAT3 and HIF1A were correlated with poor relapse-free survival. Drug–gene interaction analysis also revealed the existing pharmacological relationships that can be utilized for further experimental research. Overall, these results offer a hypothesis-generating model to understand how the gut communicates to the breast in relation to SCFAs, but do not prove a causal or therapeutic role of these events. Further experimental investigations that combine SCFA measures with molecular and clinical information are needed to confirm these relationships and elucidate their biological and translational significance.

## Data Availability

The publicly available data analyzed in this study were obtained from TCGA-BRCA (https://portal.gdc.cancer.gov/projects/TCGA-BRCA), HMDB (https://hmdb.ca/), and PubChem (https://pubchem.ncbi.nlm.nih.gov/). No new experimental datasets were generated. The processed data supporting the findings are available from the Corresponding Author on a reasonable request.
